# Supplementary material for: Mechanisms governing the pioneering and redistribution capabilities of the non-classical pioneer PU.1
Source: Nat Commun. 2020 Jan 21;11:402. doi: 10.1038/s41467-019-13960-2 (PMC6972792; doi:10.1038/s41467-019-13960-2)
Supplement: Supplementary file 7 — Source data [file 41467_2019_13960_MOESM7_ESM.zip › Source_Data/Figure6/Figure6B_MotifScanOutput/ETS-specific/homerResults.html]

/misc/data/analysis/project\_PU1/CTV1/motifs/basic/ETSSPECIFIC\_PU1mut/final - Homer de novo Motif Results


# Homer *de novo* Motif Results (/misc/data/analysis/project\_PU1/CTV1/motifs/basic/ETSSPECIFIC\_PU1mut/final)

Known Motif Enrichment Results  
Gene Ontology Enrichment Results  
If Homer is having trouble matching a motif to a known motif, try copy/pasting the matrix file into
STAMP  
More information on motif finding results: HOMER
| Description of Results
| Tips
  
Total target sequences = 7655  
Total background sequences = 40355  
\* - possible false positive  

|  |  |  |  |  |  |  |  |  |
| --- | --- | --- | --- | --- | --- | --- | --- | --- |
| Rank | Motif | P-value | log P-pvalue | % of Targets | % of Background | STD(Bg STD) | Best Match/Details | Motif File |
| 1 | C T G A T G A C G T A C A C T G A C T G C G T A C G T A C T A G A G C T T C A G T C A G A T G C | 1e-1757 | -4.047e+03 | 72.82% | 19.69% | 42.2bp (61.8bp) | Etv2(ETS)/ES-ER71-ChIP-Seq(GSE59402)/Homer(0.967)(0.981) More Information \| Similar Motifs Found | motif file (matrix) |
| 2 | C A T G C A T G G A C T C A G T A T C G G A C T C T A G A T C G A G C T G A C T C G A T | 1e-725 | -1.670e+03 | 38.35% | 10.06% | 47.3bp (60.7bp) | RUNX1(Runt)/Jurkat-RUNX1-ChIP-Seq(GSE29180)/Homer(0.977) More Information \| Similar Motifs Found | motif file (matrix) |
| 3 | G A T C A C T G A T C G C G T A C G A T C T A G G C A T T A C G T C A G | 1e-360 | -8.308e+02 | 12.50% | 1.61% | 43.9bp (57.4bp) | ETS:RUNX(ETS,Runt)/Jurkat-RUNX1-ChIP-Seq(GSE17954)/Homer(0.887) More Information \| Similar Motifs Found | motif file (matrix) |
| 4 | G T C A T C A G T A C G G T C A C G T A C T G A A G T C T A G C T C G A G T A C | 1e-352 | -8.108e+02 | 38.37% | 16.91% | 54.5bp (64.2bp) | GAMYB(MYB)/Hordeum vulgare/AthaMap(0.801) More Information \| Similar Motifs Found | motif file (matrix) |
| 5 | C G A T C A G T T G A C T A G C A C T G A C T G G A T C | 1e-340 | -7.832e+02 | 27.73% | 9.78% | 51.9bp (71.8bp) | ERT1/MA0420.1/Jaspar(0.792) More Information \| Similar Motifs Found | motif file (matrix) |
| 6 | T A C G C A T G T C G A A C G T A C T G C G T A A T C G G C A T G T A C C G T A A C G T | 1e-175 | -4.042e+02 | 9.42% | 2.14% | 52.4bp (58.2bp) | Fra1(bZIP)/BT549-Fra1-ChIP-Seq(GSE46166)/Homer(0.993) More Information \| Similar Motifs Found | motif file (matrix) |
| 7 | G A C T A G C T A T C G A G C T A C G T C G T A A C G T A G T C C G A T | 1e-140 | -3.224e+02 | 14.91% | 5.90% | 53.6bp (62.3bp) | GATA3(Zf)/iTreg-Gata3-ChIP-Seq(GSE20898)/Homer(0.984) More Information \| Similar Motifs Found | motif file (matrix) |
| 8 | C T G A G C T A C G T A C T A G G T A C G T A C C T A G A G T C | 1e-127 | -2.927e+02 | 41.05% | 27.06% | 58.1bp (66.9bp) | Bgb::run/MA0242.1/Jaspar(0.760) More Information \| Similar Motifs Found | motif file (matrix) |
| 9 | A C T G A C T G C G T A C G T A C G T A A C G T A C T G | 1e-118 | -2.737e+02 | 15.48% | 6.85% | 57.2bp (61.5bp) | AT2G31460(REMB3)/col-AT2G31460-DAP-Seq(GSE60143)/Homer(0.855) More Information \| Similar Motifs Found | motif file (matrix) |
| 10 | A G T C G A C T A C G T G A C T A T C G C G T A C G A T T A C G G C A T A C T G | 1e-110 | -2.551e+02 | 16.12% | 7.54% | 53.4bp (61.7bp) | TCF7L2/MA0523.1/Jaspar(0.947) More Information \| Similar Motifs Found | motif file (matrix) |
| 11 | G T C A T G A C G A C T G T C A G T A C T C G A G C T A G A C T A G C T G A T C G A T C G T A C | 1e-103 | -2.393e+02 | 4.74% | 0.88% | 48.7bp (67.9bp) | GFY(?)/Promoter/Homer(0.989) More Information \| Similar Motifs Found | motif file (matrix) |
| 12 | C T A G A C T G A C T G G T A C A C T G A C T G C T G A T C G A T G A C G A C T | 1e-95 | -2.200e+02 | 34.79% | 23.22% | 55.6bp (62.5bp) | KLF5/MA0599.1/Jaspar(0.884) More Information \| Similar Motifs Found | motif file (matrix) |
| 13 | C A G T T A C G G A C T C T A G C A T G T C A G C T A G C T A G G T C A C T A G A C T G T A G C | 1e-88 | -2.049e+02 | 25.77% | 15.88% | 53.5bp (60.2bp) | EGR1/MA0162.2/Jaspar(0.870) More Information \| Similar Motifs Found | motif file (matrix) |
| 14 | C T G A G T C A C T G A G T A C A C T G A T C G G T C A T G C A T C G A A T G C A T G C | 1e-78 | -1.817e+02 | 7.73% | 2.85% | 53.9bp (66.7bp) | PB0033.1\_Irf3\_1/Jaspar(0.873) More Information \| Similar Motifs Found | motif file (matrix) |
| 15 | A T G C A G C T C G T A C G T A A C G T A T G C C T G A A T C G G C A T | 1e-74 | -1.711e+02 | 13.63% | 6.99% | 56.2bp (60.3bp) | GLN3/MA0307.1/Jaspar(0.837) More Information \| Similar Motifs Found | motif file (matrix) |
| 16 | T C A G G T A C G A C T T C A G C G A T G A T C C G T A | 1e-66 | -1.524e+02 | 41.63% | 31.37% | 57.0bp (62.2bp) | MEIS2/MA0774.1/Jaspar(0.935) More Information \| Similar Motifs Found | motif file (matrix) |
| 17 | C T G A A G C T A G C T A C G T A G T C G A C T G T A C C T G A C T A G G C T A C G T A | 1e-62 | -1.444e+02 | 8.09% | 3.49% | 52.3bp (63.4bp) | STAT5(Stat)/mCD4+-Stat5-ChIP-Seq(GSE12346)/Homer(0.978) More Information \| Similar Motifs Found | motif file (matrix) |
| 18 | C T G A A G T C T C G A A C G T A G T C A G T C A C T G A G C T G A C T | 1e-61 | -1.424e+02 | 4.01% | 1.08% | 46.6bp (64.0bp) | ETV5/MA0765.1/Jaspar(0.782) More Information \| Similar Motifs Found | motif file (matrix) |
| 19 | A T C G A T G C A C G T A G C T A G C T C T A G G T A C C T A G A T C G | 1e-53 | -1.233e+02 | 12.48% | 6.97% | 54.4bp (67.3bp) | Dof2/MA0020.1/Jaspar(0.735) More Information \| Similar Motifs Found | motif file (matrix) |
| 20 | C G T A G T C A A G T C A T G C C T A G C G A T G C T A | 1e-49 | -1.143e+02 | 16.81% | 10.60% | 56.7bp (66.2bp) | MYB81(MYB)/col-MYB81-DAP-Seq(GSE60143)/Homer(0.840) More Information \| Similar Motifs Found | motif file (matrix) |
| 21 | A G T C C G A T C G T A G T C A A G T C T C A G A C T G | 1e-49 | -1.133e+02 | 30.50% | 22.46% | 56.2bp (65.1bp) | ovo/dmmpmm(Bigfoot)/fly(0.844) More Information \| Similar Motifs Found | motif file (matrix) |
| 22 | A T C G A T C G T A C G T C A G A C T G G T A C A T C G A C T G C G T A C G T A | 1e-49 | -1.129e+02 | 5.51% | 2.20% | 50.5bp (74.4bp) | RDR1/MA0360.1/Jaspar(0.867) More Information \| Similar Motifs Found | motif file (matrix) |
| 23 | C G T A A G T C A G T C A C T G A G T C C G T A A C T G | 1e-39 | -9.044e+01 | 4.78% | 1.99% | 50.8bp (67.0bp) | Bgb::run/MA0242.1/Jaspar(0.822) More Information \| Similar Motifs Found | motif file (matrix) |
| 24 | A G T C C G T A A G T C C G T A C T G A A G T C C G A T A T C G | 1e-39 | -9.006e+01 | 18.92% | 13.02% | 57.2bp (60.9bp) | Ap4(bHLH)/AML-Tfap4-ChIP-Seq(GSE45738)/Homer(0.773) More Information \| Similar Motifs Found | motif file (matrix) |
| 25 | A T G C G T A C A G C T C A T G A G T C A G C T C A T G C G T A A T C G G T C A | 1e-38 | -8.776e+01 | 6.18% | 2.97% | 54.8bp (60.0bp) | PB0205.1\_Zic1\_2/Jaspar(0.886) More Information \| Similar Motifs Found | motif file (matrix) |
| 26 | A G C T A T C G C T G A A G T C C T A G A C G T A T G C C G T A G A T C | 1e-38 | -8.752e+01 | 7.49% | 3.90% | 54.3bp (62.8bp) | FEA4(bZIP)/Corn-FEA4-ChIP-Seq(GSE61954)/Homer(0.958) More Information \| Similar Motifs Found | motif file (matrix) |
| 27 | A T C G C A G T C T A G A T G C G T C A C A G T A C T G G A T C G A C T A C T G A T C G T C A G T G C A | 1e-34 | -8.031e+01 | 2.76% | 0.87% | 55.3bp (57.5bp) | ZNF143\|STAF(Zf)/CUTLL-ZNF143-ChIP-Seq(GSE29600)/Homer(0.811) More Information \| Similar Motifs Found | motif file (matrix) |
| 28 | A T C G G A C T A G C T G T C A T G A C G A T C C G T A A G T C G C T A T C A G C T G A A T C G G C T A | 1e-34 | -7.998e+01 | 3.58% | 1.36% | 48.0bp (61.0bp) | MET28/MA0332.1/Jaspar(0.821) More Information \| Similar Motifs Found | motif file (matrix) |
| 29 | G T A C C T A G C A G T G T C A C G T A T A G C C T G A T A C G | 1e-33 | -7.684e+01 | 16.97% | 11.77% | 57.3bp (65.8bp) | ovo/dmmpmm(Bigfoot)/fly(0.827) More Information \| Similar Motifs Found | motif file (matrix) |
| 30 | A C G T C T G A A C T G A G T C G T C A C G T A A G T C | 1e-32 | -7.443e+01 | 11.63% | 7.38% | 58.0bp (64.8bp) | RFX1/MA0365.1/Jaspar(0.973) More Information \| Similar Motifs Found | motif file (matrix) |
| 31 | C G A T C A T G C T G A T G A C C G T A C T A G G T C A G C A T T G C A G C T A T G A C G C T A | 1e-31 | -7.270e+01 | 3.62% | 1.46% | 56.2bp (61.9bp) | GATA2/MA0036.2/Jaspar(0.748) More Information \| Similar Motifs Found | motif file (matrix) |
| 32 | A G T C T A C G A T C G A T G C G C A T T A G C A G C T A G C T A G C T G A T C T A G C A T C G A T C G | 1e-30 | -6.935e+01 | 2.06% | 0.59% | 47.6bp (68.6bp) | Pnt/dmmpmm(Papatsenko)/fly(0.711) More Information \| Similar Motifs Found | motif file (matrix) |
| 33 | A G T C C G A T C A G T C T G A G C A T A G T C G C A T A T C G G T C A C G A T T A G C G C T A | 1e-30 | -6.935e+01 | 1.57% | 0.34% | 52.2bp (58.0bp) | Gata1(Zf)/K562-GATA1-ChIP-Seq(GSE18829)/Homer(0.797) More Information \| Similar Motifs Found | motif file (matrix) |
| 34 | T C G A C G A T T G A C G T A C A C T G T A G C A G T C A T G C A G C T G C T A | 1e-27 | -6.326e+01 | 2.53% | 0.90% | 54.0bp (67.8bp) | PB0143.1\_Klf7\_2/Jaspar(0.771) More Information \| Similar Motifs Found | motif file (matrix) |
| 35 | C T A G G T A C C T A G A G T C G C T A C A G T A C T G A G T C A C T G A G T C T G C A C T A G | 1e-26 | -6.172e+01 | 3.06% | 1.23% | 55.7bp (64.7bp) | NRF1(NRF)/MCF7-NRF1-ChIP-Seq(Unpublished)/Homer(0.974) More Information \| Similar Motifs Found | motif file (matrix) |
| 36 | A G T C A G T C C T G A T A G C G A C T C T G A T A C G T C G A A C T G T C A G T A C G G T A C T C A G A T G C | 1e-25 | -5.829e+01 | 1.63% | 0.44% | 57.8bp (63.8bp) | BORIS(Zf)/K562-CTCFL-ChIP-Seq(GSE32465)/Homer(0.882) More Information \| Similar Motifs Found | motif file (matrix) |
| 37 | T A G C T C G A T C G A T C G A T G C A G C A T C A T G C A T G T G A C A C T G A T C G A G T C | 1e-23 | -5.420e+01 | 2.56% | 1.01% | 56.3bp (62.1bp) | YY1(Zf)/Promoter/Homer(0.946) More Information \| Similar Motifs Found | motif file (matrix) |
| 38 | C A G T T A G C C T G A T G C A G T C A C G T A T C A G T A G C G T A C | 1e-21 | -4.966e+01 | 3.93% | 1.99% | 56.4bp (62.6bp) | Sf3b4(RRM)/Danio\_rerio-RNCMPT00224-PBM/HughesRNA(0.840) More Information \| Similar Motifs Found | motif file (matrix) |
| 39 | A T G C C T A G G T A C C A T G A C T G C A G T A C T G A T G C G T C A C A G T C A G T A T G C A G C T A C T G | 1e-20 | -4.748e+01 | 0.81% | 0.13% | 57.4bp (54.3bp) | ABI4(2)(AP2/EREBP)/Zea mays/AthaMap(0.696) More Information \| Similar Motifs Found | motif file (matrix) |
| 40 | C T A G T A C G T G A C C G T A A C T G A G C T G A C T C T A G A G T C G A C T C T G A | 1e-20 | -4.636e+01 | 3.15% | 1.49% | 55.3bp (61.0bp) | PB0149.1\_Myb\_2/Jaspar(0.812) More Information \| Similar Motifs Found | motif file (matrix) |
| 41 | C T G A C T G A C T G A A C T G A C T G G T C A C T G A T C G A G T A C G C T A A T C G A G T C G A C T A C T G | 1e-20 | -4.630e+01 | 0.59% | 0.06% | 45.7bp (53.6bp) | ETS:E-box(ETS,bHLH)/HPC7-Scl-ChIP-Seq(GSE22178)/Homer(0.914) More Information \| Similar Motifs Found | motif file (matrix) |
| 42 | A G T C A C T G T A C G C G A T C G A T A C T G A G T C G A C T T G C A A C G T C A T G C T A G | 1e-19 | -4.467e+01 | 1.67% | 0.57% | 58.5bp (64.1bp) | PB0055.1\_Rfx4\_1/Jaspar(0.885) More Information \| Similar Motifs Found | motif file (matrix) |
| 43 | A T C G A C G T G A C T C A T G G T A C T A C G C G A T T G A C T C G A | 1e-19 | -4.388e+01 | 2.27% | 0.95% | 55.4bp (60.6bp) | RTG3(MacIsaac)/Yeast(0.802) More Information \| Similar Motifs Found | motif file (matrix) |
| 44 | T C G A T A C G T A G C T A C G A T C G T C G A G C T A C T A G T A C G T A C G C T A G T A C G A G T C A T C G | 1e-18 | -4.205e+01 | 1.38% | 0.43% | 57.0bp (66.8bp) | PB0076.1\_Sp4\_1/Jaspar(0.728) More Information \| Similar Motifs Found | motif file (matrix) |
| 45 | A G T C A G T C C G T A G T C A A G C T T A G C T C G A T A C G G T C A C T G A A T C G T G A C G A T C C T A G | 1e-17 | -4.054e+01 | 0.91% | 0.20% | 52.7bp (63.7bp) | HAP5/MA0316.1/Jaspar(0.818) More Information \| Similar Motifs Found | motif file (matrix) |
| 46 | G A C T C T A G G T A C C A T G G A T C G C T A C T G A | 1e-16 | -3.863e+01 | 7.56% | 5.07% | 57.0bp (71.6bp) | SWI6/SWI6\_YPD/9-SWI4,9-SWI6(Harbison)/Yeast(0.806) More Information \| Similar Motifs Found | motif file (matrix) |
| 47 | A C G T T G A C A G C T G A T C T A C G A T G C T A C G T G C A T C A G T G C A A C T G A G C T | 1e-16 | -3.861e+01 | 0.90% | 0.21% | 56.8bp (61.0bp) | GFX(?)/Promoter/Homer(0.965) More Information \| Similar Motifs Found | motif file (matrix) |
| 48 | C G T A T A C G G A T C A G C T T G A C A C G T A C G T A C G T C T G A C A G T T A G C G A C T | 1e-15 | -3.624e+01 | 0.39% | 0.03% | 63.4bp (37.3bp) | PH0152.1\_Pou6f1\_2/Jaspar(0.706) More Information \| Similar Motifs Found | motif file (matrix) |
| 49 | A C T G C G T A C A G T A C T G G T A C A G T C G C T A A T C G C G T A A C T G C G T A A C G T C G T A C T G A | 1e-12 | -2.857e+01 | 0.33% | 0.02% | 41.4bp (36.8bp) | Gata4/MA0482.1/Jaspar(0.670) More Information \| Similar Motifs Found | motif file (matrix) |
| 50 | G A C T T A G C A T G C C A T G A C T G T C G A T C G A C T A G T A G C A G T C T C G A T A G C A C T G A T G C | 1e-12 | -2.828e+01 | 0.61% | 0.13% | 46.0bp (42.1bp) | PUT3(MacIsaac)/Yeast(0.686) More Information \| Similar Motifs Found | motif file (matrix) |
